# MicroRNA-7, synergizes with RORα, negatively controls the pathology of brain tissue inflammation

**DOI:** 10.1186/s12974-020-1710-2

**Published:** 2020-01-20

**Authors:** Dongxu Yue, Juanjuan Zhao, Huizi Chen, Mengmeng Guo, Chao Chen, Ya Zhou, Lin Xu

**Affiliations:** 1 Special Key Laboratory of Gene Detection & Therapy of Guizhou Province, Zunyi, 563099 Guizhou China; 2 Department of Immunology, Zunyi Medical University, Zunyi, 563099 Guizhou China; 3 Department of Medical Physics, Zunyi Medical University, Zunyi, 563099 Guizhou China

**Keywords:** miR-7, Brain tissue inflammation, Neuron, NF-κB, RORα, RNA interference

## Abstract

**Background:**

Accumulating evidence has documented that microRNA-7 (miR-7) plays an important role in the pathology of various diseases. However, the potential role of miR-7 in brain tissue inflammation (BTI) remains unclear.

**Methods:**

We detected the expression of miR-7 in LPS-induced murine BTI model and observed the possible effects of miR-7 deficiency on the pathology of BTI. To elucidate the mechanism, the target gene of miR-7 was screened out by Gene chip assay and its potential roles in BTI were evaluated by Western blot, immunofluorescence, and RNAi assay, respectively.

**Results:**

MiR-7 was upregulated in brain tissue in BTI mice and its deficiency could significantly aggravate the pathology of brain tissue. Moreover, RORα, a new target molecule of miR-7, was upregulated in brain tissue from miR-7 deficiency BTI mice. Of note, downregulation of RORα could remarkably exacerbate the pathology of brain tissue and elevate the transduction of NF-κB and ERK1/2 signaling pathways in brain tissue from miR-7 deficiency BTI mice. Furthermore, RORα and miR-7 were dominantly co-expressed in neurons of BTI mice. Finally, RORα synergized with miR-7 to control the inflammatory reaction of neuronal cells in response to LPS stimulation.

**Conclusions:**

MiR-7 expression is upregulated in BTI model. Moreover, miR-7 synergizes with its target gene RORα to control the inflammation reaction of neurons, thereby orchestrating the pathology of BTI.

## Background

Brain tissue inflammation (BTI), a complicated process including a series of integrated steps, is closely related to the occurrence and development of various brain diseases [1–3]. Up to now, the underlying mechanism of pathology of BTI remains elusive. Recent evidence has shown that microRNAs (miRNAs), small endogenous RNAs of 21–25 nucleotides capable of guiding the post-transcriptional silencing of their target mRNAs through base pairing encompassing mature mRNA 3′-untranslated region (3′-UTR), play essential regulatory roles in the development of BTI [4–6]. For instance, miR-126 is implicated in brain vascular inflammation and intracerebroventricular (I.C.V) administration of miR-126-3p mimic can significantly suppress the upregulation of phosphoinositide-3-kinase regulatory subunit 2, as well as reduce blood-brain barrier permeability and brain edema [7]. Moreover, Peli1 is an important E3 ubiquitin-protein ligase contributing to neuroinflammation. MiR-142a-3p and miR-155-5p can directly suppress Peli1 expression and protect against the inflammatory effects of METH treatment partially through activating p38 MAPK and NF-κB inflammatory pathways [8]. These studies indicate that miRNAs might be potential candidates for therapy against BTI. Therefore, further investigation on the possible roles of distinct miRNA molecules in the development of BTI will not only benefit to the understanding on pathology of BTI, but also be valuable for the development of novel therapeutic strategies against brain diseases.

MicroRNA-7 (miR-7) was first identified by Lagos-Quintana in 2001 [9] and has been reported to regulate the biology of various tumor cells and the development of inflammation diseases by repressing the expression of different target molecules [10, 11]. For instance, Fan et al. found that miR-7 suppressed angiogenesis of colorectal cancer cells through ERK signaling by downregulation epidermal growth factor receptor [12]. Moreover, Ye et al. reported that miR-7 deficiency promoted p65-mediated aberrant NF-κB activation to facilitate gastric cancers metastasis and ultimately resulted in the worse clinical outcome in human gastric cancer [13]. Our previous study also showed that miR-7 regulated TLR9 signaling and affected the growth and metastatic potential of human lung cancer cells [14]. Importantly, many recent studies have reported that miR-7 is dominantly expressed in brain tissue and involved in the biological functions of brain [15–17]. Moreover, miR-7 also plays an important role in the development of brain diseases [18–20]. For example, Chen et al. reported that miR-7 was closely related to the differentiation of neural stem cells and the development of the cerebral cortex [21]. Moreover, Kabaria et al. found that miR-7 exerted a cytoprotective effect by elevating the expression level of Nrf2 through inhibiting Keap1 expression [22]. However, the exact role of miR-7 involved in the development of BTI has yet to be fully elucidated.

To this aim, in the present study, we analyzed the expression level of miR-7 in LPS-induced murine BTI model and observed the possible influence of miR-7 deficiency on the pathology of BTI. We found that miR-7 was upregulated in brain tissue in BTI model and its deficiency could significantly aggravate the development of brain inflammation. Moreover, RORα, a new target molecule of miR-7, was upregulated in brain tissue from miR-7 deficiency BTI mice. Of note, we found that downregulation of RORα could remarkably exacerbate the pathology of brain tissue, accompanied by elevated transduction of NF-κB and ERK1/2 signaling pathways in miR-7 deficiency BTI mice. Thus, our data suggested a novel network model in which miR-7 synergizes with, but not antagonizes, its target gene RORα to control the pathology of BTI, which could ultimately aid the understanding of the pathogenesis of BTI and the development of new therapeutic strategies against clinical inflammatory brain diseases.

## Materials and methods

### Mice

C57BL/6 wild-type (WT) mice and C57BL/6 background miR-7 deficiency (miR-7^*def*^) mice (both 8–10 weeks of age) were housed under specific pathogen-free (SPF) conditions at Zunyi Medical University [23].

### LPS-induced murine BTI model

WT mice andmiR-7^*def*^ mice were intraperitoneally injected with LPS (2.5 mg/kg of body weight, *Escherichia coli* 0111: B4; Sigma) and control group with vehicle (PBS) [24]. After 12 h, the brain tissue was collected.

### Histopathology

Brain tissue was fixed in 4% (w/v) paraformaldehyde, followed by routine dehydration and embedded in paraffin, and cut into 4 μm thick. Then, slices were subjected to xylene dewaxing, gradient ethanol dehydration, routine hematoxylin-eosin staining (H&E), and again dehydrated with gradient ethanol, followed by xylene transparency and mounting. Pathological changes in brain tissue were observed under a light microscope (Olympus, Tokyo, Japan). Two investigators blinded to group assignments analyzed the samples and determined levels of brain inflammation injury.

### Quantitative real-time PCR analysis

For miR-7 expression analysis, cDNA was synthesized by TaqMan MicroRNA Reverse Transcription Kit (ThermoFisher Scientific) using S1000TM Thermal cycler PCR Amplifier (Bio-Rad). Next, real-time PCR was performed to quantify miR-7 expression by miR-7 probe of TaqMan (Life Technologies) according to the instructions of the manufacturer using C1000TM Thermal cycler Quantitative Real-time PCR Amplifier (Bio-Rad). U6 as endogenous control was used for normalization. For mRNA analysis, cDNA was synthesized using a Prime Script RT reagent kit (Takara, Kusatsu, Japan) according to the manufacturer’s instructions. Real-time PCR was carried out by SYBR Premix Ex Taq II (Takara, Kusatsu, Japan) according to the instructions of the manufacturer. GAPDH as endogenous control was used for normalization. The relative expression levels of miR-7 and mRNAs were determined using the standard 2^−ΔΔCT^ (cycle threshold) method. The sequences of the primers used for real-time PCR were shown in Table 1.

**Table 1:** The primer sequence used for real-time quantitative PCR

Gene	Primer sequence (5′-3′)
TGF-β	F: CCCCATTCCTACTTCTCC
	R: ACGCACCTTTCTGGTTACAC
IL-6	F: AGACAAAGCCAGAGTCCTTCAG
	R: GGTCTTGGTCCTTAGCCACTC
TNF-α	F: TGTCTACTGAACTTCGGGGTG
	R: CTGCTCCTCCACTTGGTGGTT
IL-1β	F: GAGCTTCAGGCAGGCAGTAT
	R: TTGTTCATCTCGGAGCCTGTA
TROVE2	F: GAAGTGTGTCGCATTCCGACC
	R: GGACAGTCGGAGGAGATCTTT
RORα	F: GGAGACAAATCGTCAGGAATC
	R: ACCAAACTTGACAGCATCTCG
Col4a3bp	F: TTGAAGCTGCTCTTGACAGAC
	R: TCTATGCGTCCCGACAGAAGA
Gpr158	F: AATTAGAAGCAGCCCAATGG
	R: TTTCACGAACAGCACAAAGAA
Zfp212	F: ACCAAGTCACCCACCATCTCT
	R: GAGAACCTGCTTCGAAACAA
Socs4	F: CCCCAGTGCCTGTATGTTCTT
	R: CTGCTGCTCTGGCACATCAAT
C2cd2	F: TCAGGCCTTAGCCATGTGT
	R: CGTGGGGACTTGAGTTTCA
Syt4	F: GAGAAGCTGGGGACACTCTT
	R: ACGGGTCAGAGGTCATGGATT
Spock3	F: CAGTCTGTGGTTCTGATGGGC
	R: GACTTATCGGAGGGACATGG
Gpr22	F: GCAAAACACCAACTGCTCCAA
	R: ACTGCATGTTGATTTCCAGAA
Basp1	F: CTCTTTGACGGCCACGCTTTG
	R: CTGAGCAAGAAGAAGAAGGGCT
Tmed5	F: TCGATCTCCAACGATGCCTT
	R: TCACACCCTCTTTGGACAGTG
Ptprj	F: TGCCATTTGCATTGCTCCAG
	R: CTTAAGCCCAGGCACTTCGT
Zfp606	F: TCATGGACCAGTCTTGGGGG
	R: ATCTCACCTCGACTTGGGCTA
Aqp11	F: TGCAGGAATCCCATCCACAC
	R: CCCTCCTGCATAGGCCAAAA
1110059G10Rik	F: TCTTCAGCTGTTAGGTCTCCC
	R: GAGCAAGCGGAACCAAGTGT
Gtdc1	F: CACCCTTCTGTGTGGAGCTGA
	R: TTACGGGGTAAGTAGCCCCA
GAPDH	F: GAAGGTCGGAGTCAACGGATT
	R: ATGGGTGGAATCATATTGGAA
miR-7	F: CGGCGGTGGAAGACTAGTGATT
U6	F: AGAGAAGATTAGCATGGCCCCTG
Common reverse	R: ATCCAGTGCAGGGTCCGAGG

Note: *TROVE2* TROVE domain family, member 2; *RORα* RAR-related orphan receptor alpha; *Col4a3bp* collagen, type IV, alpha 3 (Goodpasture antigen) binding protein; *Gpr158* G protein-coupled receptor 158; *Zfp212* Zinc finger protein 212; *Socs4* suppressor of cytokine signaling 4; *C2cd2* C2 calcium-dependent domain containing 2; *Syt4* synaptotagmin IV; *Spock3* sparc/osteonectin, cwcv and kazal-like domains proteoglycan 3; *Gpr22* G protein-coupled receptor 22; *Basp1* brain abundant, membrane attached signal protein 1; *Tmed5* transmembrane emp24 protein transport domain containing 5; *Ptprj* protein tyrosine phosphatase, receptor type, J; *Zfp606* zinc finger protein 606; *1110059G10Rik* RIKEN cDNA 1110059G10 gene; *Gtdc1* glycosyltransferase-like domain containing 1; *F* forward primer; *R* Reverse primer

### Fluorescence in situ hybridization (FISH)

To evaluate the cellular distribution of pre-miR-7-2 in the brain, FISH assay was performed based as our previous description with some modifications [23]. Briefly, before hybridization incubation, all solutions were prepared with diethylpyrocarbonate-treated water. After deparaffinization and rehydration, tissue sections were treated by pepsin digestion. Sections were next incubated or heated in the microwave, and then were incubated with hybridization cocktail containing miR-7-2 probe (1:1000; EXIQON; no. 38485–01) at 42 °C for overnight. Next, the sections were washed in PBS and incubated with a secondary antibody of Cy3 conjugated goat-anti-rabbit IgG (1:250; Invitrogen) in the dark, at room temperature for 1 h. Then, the slides were rinsed with PBS-T three times, for 5 min each and counterstained, mounted with Slow Fade Gold Antifade Reagent with DAPI (1:1000) in the dark, at room temperature for 10 min, before examination by fluorescence microscopy (Zeiss Axioplan 2).

### Enzyme-linked immunosorbent assay (ELISA)

The brain tissue was homogenized in 100 mg/mL cold PBS. The samples were centrifuged at 14,000×*g* for 15 min. The brain tissue supernatant or neuronal PC12 cells culture supernatant was collected for a protein assay using a BCA protein assay reagent kit (Solarbio, Beijing, China). The concentration of IL-1β, IL-6, TNF-α, and TGF-β were determined using Quantikine Immunoassay kit (eBioscience) according to the manufacturer’s instructions, respectively. The ELISA results were normalized to total protein concentration.

### Multiplexed fluorescent immunohistochemical staining

According to Opal protocol of multiplexed fluorescent staining, slides were deparaffinized in xylene and rehydrated in ethanol. Antigen retrieval was performed in 10 mmol/L citric acid buffer (pH 6.0) for 10 min using a 750-W microwave and rinsed with PBS-T three times, for 5 min each, slides were blocked with 10% normal goat serum at room temperature for 30 min and then, incubated with rabbit anti-mouse RORα antibody (1:400; Abcam; no. ab60134) overnight at 4 °C. After the overnight incubation, slides were rinsed with PBS-T three times and incubated with Goat anti-Rabbit IgG H&L (HRP) secondary antibody (1:1000 dilution; Abcam; no. ab6721) at room temperature for 1 h. Later, RORα was visualized using PPD520 tyramine signal amplification Plus (1:100). Subsequently, slides were placed in citrate buffer (pH 6.0) and subjected to microwave again, and then incubated with primary rabbit antibodies for IBA-1 (1:1000 dilution; Abcam; no. ab178847) in a humidified chamber at room temperature for 1 h. After incubating with Goat Anti-Rabbit IgG H&L (HRP) secondary antibody for 1 h. IBA-1 was then visualized using PPD570 tyramine signal amplification. After washing in PBS three times, sections were counterstained, mounted with Slow Fade Gold Antifade Reagent with DAPI, and left for 10 min in the dark at room temperature before examination by fluorescence microscopy.

### RORα RNAi transfer experiments in vivo

According to previous reports [25–27], the stereotaxic coordinates were 0.8 mm posterior, 1.2 mm lateral to the bregma, and 3 mm ventral to the surface of the skull. Twelve mice were divided randomly into two groups (*n* = 6 per group), 5 μL RORα siRNA or negative control (NC) siRNA was diluted with the same volume of transfection reagent in vitro (Invivofectamine 3.0; ThermoFisher, Scientific). After mixing gently, the solution was I.C.V. through a micro syringe according to the guidance of stereotaxic instrument (Kent Scientific Co., Torrington, CT, USA) under anesthetized.

### Cell culture

Neuronal PC12 cells and human embryonic kidney cell line HEK 293T cells (saved in our lab) were cultured in completed Roswell Park Memorial Institute (RPMI-1640; GIBCO) medium containing 10% (v/v) FBS, penicillin (100 IU/mL), and streptomycin (100 μg/mL) on 100-mm dishes at 37 °C under 5% CO_2_ humidified atmosphere.

### Plasmid construction and luciferase reporter assay

The luciferase reporter assay was performed to determine whether RORα (Gene ID, 1988338) was a direct target of miR-7. The possible sites (1806 bp–1812 bp segment, ACTTGTT) of binding between RORα 3′-UTR and miR-7 were predicted using miRDB database (http://www.mirdb.org). A fragment of 200 bp containing wild-type RORα 3′-UTR (5′-ACTTGTT-3′) or a random mutation sequence of mutant RORα 3′-UTR (5′-GTCCACC-3′) was directly synthesized (Sangon, Shanghai, China). Two fragments were ligated to pEZX-FR02 reporter vector (GeneCopoeia, Rockville, MA, USA), respectively. Then, the wild-type (RORα 3′-UTR WT) or mutant reporter vector (RORα 3′-UTR MUT) was co-transfected into HEK-293T cells in 12-well plates with 100 nm miR-7 mimics or negative control (NC) mimics by Lipofectamine 3000 (Invitrogen), respectively. After 48 h, cells were lysed and subjected to luciferase assays using the Dual Luciferase Reporter Assay System (Promega).

### Tissue immunohistochemistry (IHC)

Immunohistochemical staining was done using the SP method (universal immunohistochemical staining kit, Zhong-shan Golden Bridge BioTechnology Beijing, China). Slices were subjected to xylene dewaxing and gradient ethanol dehydration. Antigen retrieval was performed in 10-mmol/L citric acid buffer (pH 6.0) for 10 min using a 750-W microwave and incubated with 3% (v/v) methanol-hydrogen peroxide to block endogenous peroxidases at room temperature for 15 min. Next, slices were incubated with rabbit anti-mouse antibodies at appropriate dilution in TBST overnight at 4 °C. The primary antibodies used were as follows: IBA-1 (1:8000; Abcam; no. ab178847), GFAP (1:500; Abcam; no. ab68428), NeuN (1:1000; Abcam; no. ab177487), RORα (1:1000; Abcam; no. ab60134), and NF-κB (1:1000; Abcam; no. ab32536). PBS instead of primary antibody served as a control. Slices were rinsed with PBS-T three times and incubated with Goat Anti-Rabbit IgG H&L (HRP) secondary antibody (1:1000; Abcam; no. ab6721) at room temperature for 1 h. Finally, slices were incubated with streptavidin-biotin protein and peroxidase (1:200) and counterstained with hematoxylin before observed under a light microscope.

### Western blotting analysis

After brain tissue was homogenates, total protein was extracted, and protein concentration was quantified using the BCA Protein Quantitation Kit. Total protein was mixed with 5 × SDS protein sample buffer solution (4:1) and heated at 100 °C for 10 min and stored at − 20 °C. Protein samples were subjected to SDS-PAGE and transferred to polyvinylidene difluoride (PVDF) membranes (Bio-Rad). The membranes were blocked with 5% skim milk in PBS plus 0.05% Tween 20 (PBS-T) at room temperature for 90 min, and then incubated with rabbit anti-mouse antibodies at appropriate dilution in TBST overnight at 4 °C. The primary antibodies used were as follows: RORα (1:1000; Abcam; no. ab60134), NF-κB (1:1000; Cell Signaling Technology; no. 4764), phos-NF-κB (1:1000; Cell Signaling Technology; no. 3039), ERK (1:1000; Cell Signaling Technology; no. 4695), phos-ERK (1:1000; Cell Signaling Technology; no. 4370), AKT (1:1000; Cell Signaling Technology; no. 4691), phos-AKT (1:1000; Cell Signaling Technology; no. 4060), and GAPDH (1:5000; Abcam; no. ab8245). PBS instead of primary antibody served as a control. After overnight incubation, the membrane was rinsed with PBS-T three times and incubated with Goat Anti-Rabbit IgG H&L (HRP) secondary antibody (1:2000; Abcam; no. ab6721) at room temperature for 1 h. Finally, the signals were determined by chemiluminescence image system (Bio-Rad).

### Immunofluorescence (IF)

Sections were hydrated and rinsed with PBS three times, for 5 min each, then blocked with 10% normal goat serum at room temperature for 30 min and incubated with rabbit anti-mouse antibodies at appropriate dilution in TBST overnight at 4 °C. The primary antibodies used were as follows: RORα (1:100; Abcam; no. ab60134), NeuN (1:100; Abcam; no. ab177487), and GFAP (1:100; Cell Signaling Technology; no. 80788). PBS instead of primary antibody served as a control. Then, slices were rinsed with cold PBS three times, for 5 min each, and incubated with a secondary antibody of Alexa Fluor 488-conjugated Goat Anti-Rabbit IgG (1:500; Invitrogen), Cy3-labeled Goat Anti-Rabbit IgG (H+L) (1:500; Beyotime) in the dark, at room temperature for 1 h. Finally, sections were mounted with Slow Fade Gold Antifade Reagent with DAPI, and examination by fluorescence microscopy (Zeiss Axioplan 2).

### Gene expression microarray

Global gene expression array data was available in the National Center for Biotechnology Information (NCBI) Gene Expression Omnibus (GEO) under accession number GEO: GSE122114.

### Nissl staining

Brain tissue was fixed in 4% (w/v) paraformaldehyde, paraffin-embedded, and sliced in sections of 4-μm thickness. After dewaxing in xylene and rehydration through graded ethanol, the sections were hydrated in 1% (w/v) toluidine blue at 50 °C for 20 min. Two investigators blinded to group assignments analyzed the samples and determined the level of brain inflammation injury. All brain fields at original magnification × 100 and × 400 were examined for each example.

### Statistical analyses

Data were expressed as the mean ± SEM from at least three independent experiments. Statistical analysis was performed by unpaired Student’s test (two-tailed) for two groups Student’s *t* test or one-way analysis of variance (ANOVA) with Bonferroni’s correction for three or more groups to evaluate statistical significance using GraphPad Prism 7.0 software. *P* < 0.05 was considered statistically significant.

## Results

### MiR-7 is upregulated in brain tissue of LPS-induced murine BTI model

**Fig. 1:**
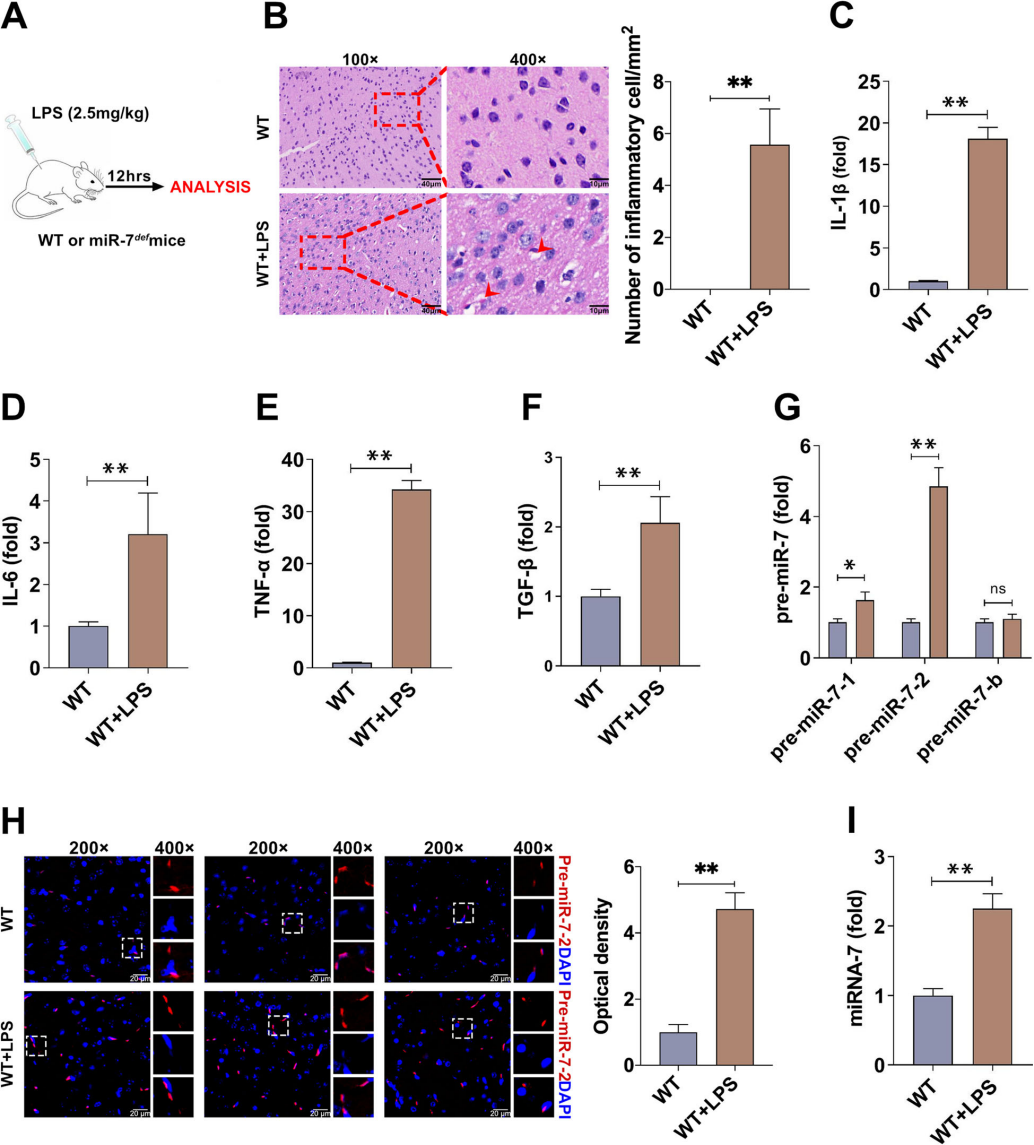
The expression of miR-7 is upregulated in BTI model. **a** Schematic representation of the animal experiments. **b** WT mice (*n* = 6 per group) were intraperitoneally injected with LPS (2.5 mg/kg of body weight) and control group with PBS. After 12 h, the pathology of brain tissue was observed by H&E staining (*n* = 3 per group; arrows indicate hemorrhage foci). **c**–**f** The relative expression levels of cytokines (IL-1β, IL-6, TNF-α, and TGF-β) were detected by real-time PCR assay and calculated (*n* = 6 per group; *t* test). **g** The expression levels of pre-miR-7-1, pre-miR-7-2, and pre-miR-7-b in brain tissue were determined by real-time PCR assay and calculated (*n* = 6 per group; *t* test). **h** The expression level of pre-miR-7-2 in brain tissue was determined by FISH (*n* = 3 per group). **i** The relative expression of mature miR-7 in brain tissue was analyzed by real-time PCR assay and calculated (*n* = 6 per group, *P < 0.05, **P < 0.01)

We first tested the expression level of miR-7 in BTI model. As shown in Fig. 1 a and b, inflammatory cell infiltration and hemorrhage foci were observed in brain tissue of murine BTI model. Moreover, the relative expression levels of inflammatory cytokines IL-1β, IL-6, TNF-α, and TGF-β in brain tissue also increased significantly (Fig. 1c–f, *P* < 0.05), indicating BTI model was successfully constructed [24]. As shown in Fig. 1 g, Real-time PCR assay showed that the relative expression levels of pre-miR-7-1 and pre-miR-7-2 increased obviously, especially for pre-miR-7-2. Next, we further determined the expression of pre-miR-7-2 in brain tissue by FISH assay and obtained similar results (Fig. 1h). Importantly, the relative expression level of mature miR-7 also increased significantly in brain tissue in murine BTI model (Fig. 1i, *P* < 0.05). These results demonstrate that miR-7 is upregulated in brain tissue in BTI model.

### MiR-7 deficiency aggravates the pathology of BTI

**Fig. 2:**
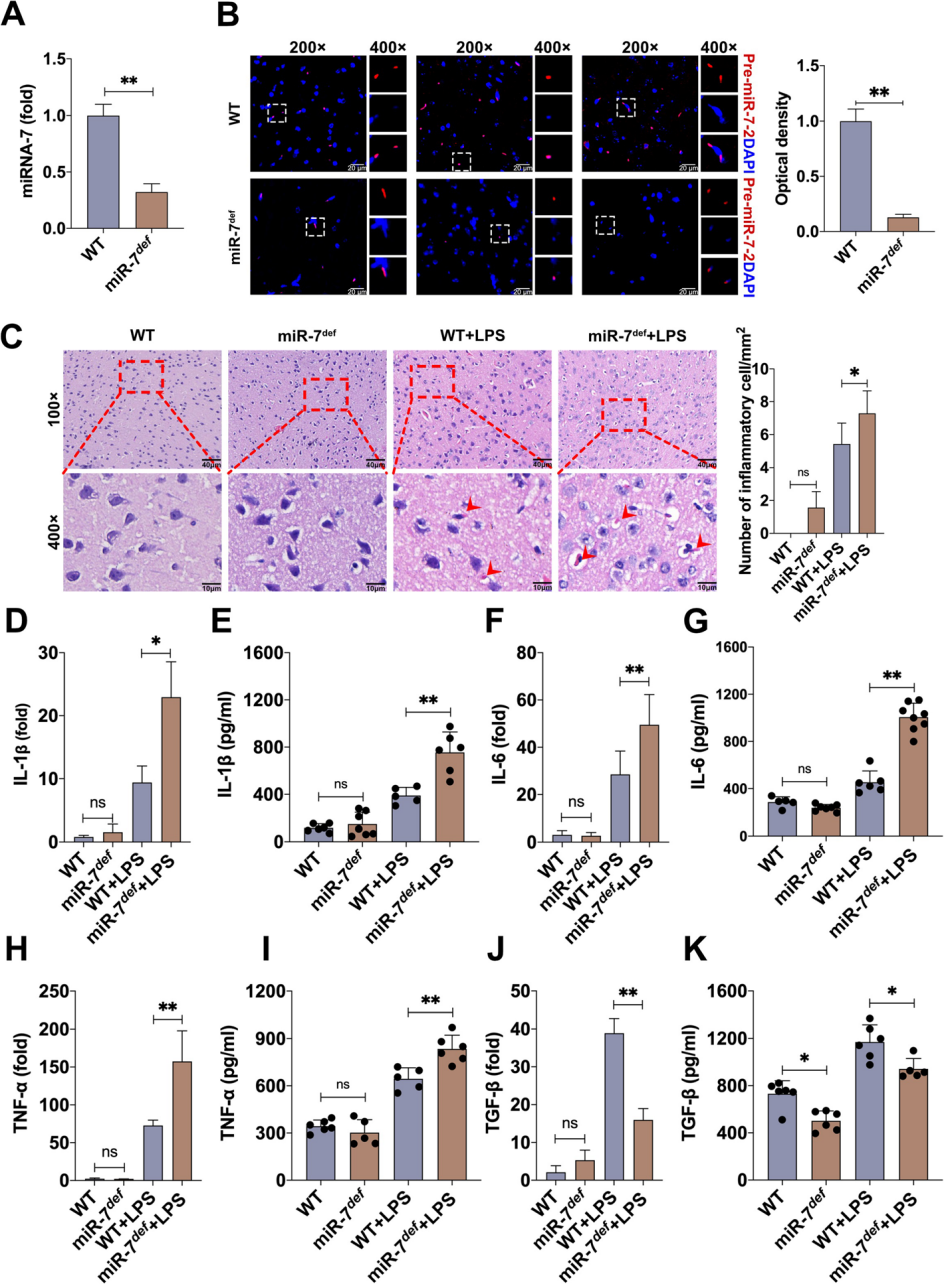
MiR-7 deficiency aggravates the pathology of BTI model. Animal experiments were performed according to Fig. 1a. **a** The expression level of miR-7 in brain tissue was determined by Real-time PCR assay and calculated, respectively. **b** The expression level of pre-miR-7-2 was determined by FISH (*n* = 4 per group). WT mice and miR-7^*def*^ mice were intraperitoneally injected with LPS and control group with PBS. After 12 h, **c** the pathology of brain tissue was observed by H&E staining (*n* = 3 per group). Arrows in **c** indicate hemorrhage foci. **d**–**k** The expression levels of IL-1β, IL-6, TNF-α, and TGF-β were analyzed by real-time PCR and ELISA assay (*n* = 6 per group; one-way ANOVA, *P < 0.05, **P < 0.01)

Next, we further investigated the potential role of miR-7 in the pathology of BTI. As shown in Fig. 2 a and b, the expression level of miR-7 significantly decreased in the brain tissue of miR-7^*def*^mice, which was consistent with our previous work [23]. Importantly, compared with WT BTI model, the number of inflammatory cells and hemorrhage foci increased obviously the brain tissue of miR-7^*def*^ BTI model (Fig. 2c). Real-time PCR analysis further showed that the expression levels of pro-inflammatory factor IL-1β, IL-6, and TNF-α in brain tissue also increased significantly in miR-7^*def*^BTI model (Fig. 2d, f, h, *P* < 0.05). By contrast, the expression level of anti-inflammatory factor TGF-β decreased noticeably (Fig. 2j, *P* < 0.05). Meanwhile, similar results were obtained by ELISA assay (Fig. 2e, g, i, k, *P* < 0.05). These data indicate that miR-7 deficiency aggravates the pathology of BTI.

**Fig. 3:**
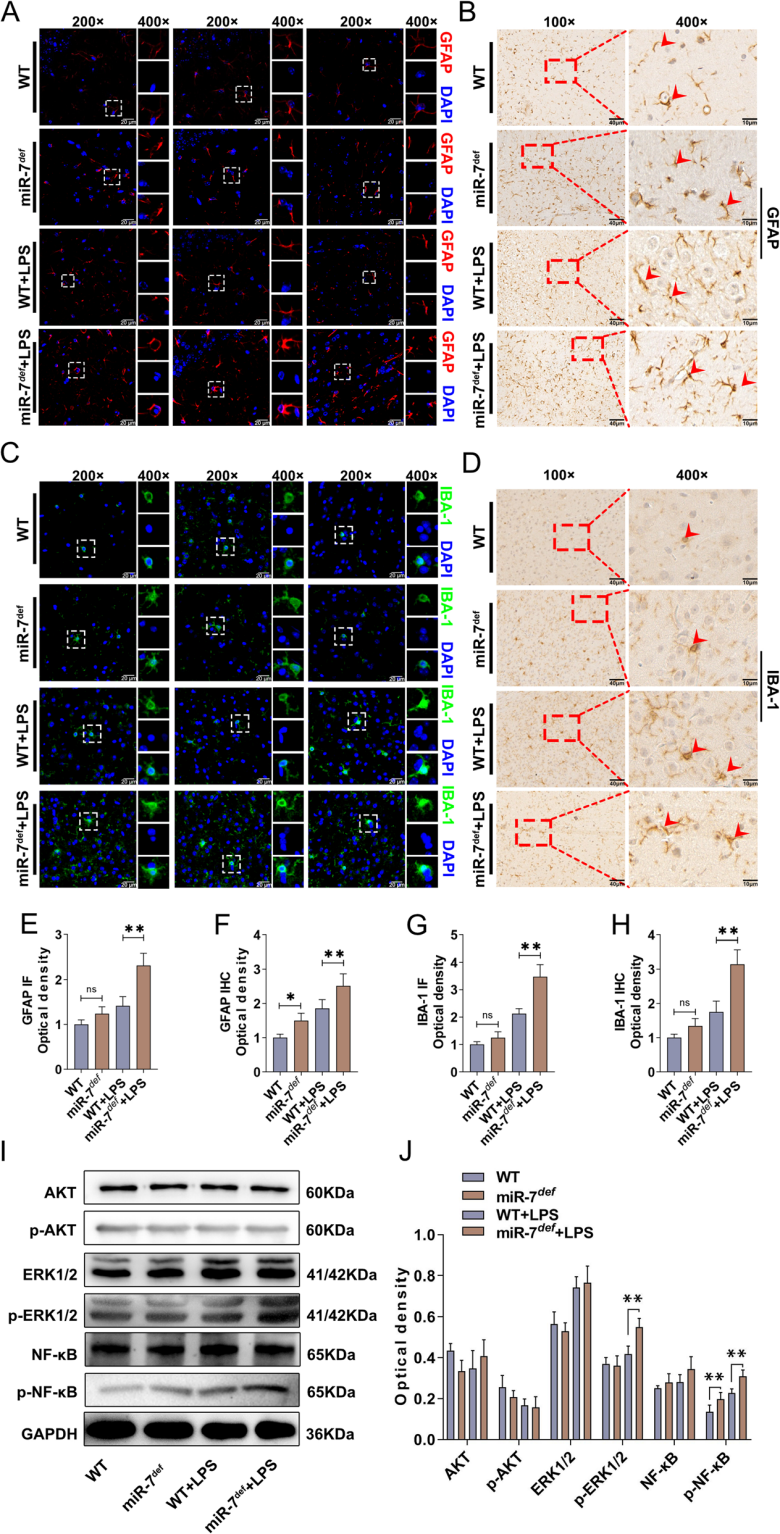
MiR-7 deficiency alters the composition of immune cells and the transduction of NF-κB, ERK signaling pathways in BTI model. WT mice and miR-7^*def*^ mice were intraperitoneally injected with LPS (2.5 mg/kg of body weight) and control group with PBS. After 12 h, **a**–**h** the number of astrocytes in hippocampus and microglia in cerebral cortex were analyzed by IF and IHC and calculated (*n* = 3, one-way ANOVA, **P < 0.01). **i**, **j** The protein levels of AKT, phos-AKT, ERK1/2, phos-ERK1/2, NF-κB, and phos- NF-κB in brain tissue were analyzed by Western blot assay and calculated, respectively (*n* = 3, one-way ANOVA, **P < 0.01)

It is well known that astrocytes and microglia play an important role in brain inflammation [28–30]. To further investigate the effect of miR-7 deficiency on the pathology of BTI, we observed the possible changes on astrocytes and microglia in brain tissue and found that the number of astrocytes and microglia were elevated in miR-7^*def*^ BTI model (Fig. 3a–h, *P* < 0.05). Previous literatures have demonstrated that some signaling pathways, including AKT, ERK1/2, and NF-κB signaling pathways, were involved in the development of inflammatory diseases [31–33]. Thus, to elucidate whether miR-7 deficiency resulted in the change on these signaling pathways, the expression levels of AKT, phos-AKT, ERK1/2, phos-ERK1/2, as well as NF-κB and phos-NF-κB, were analyzed, respectively. As shown in Fig. 3 i and j, compared with those in WT BTI model, the expression levels of phos-NF-κB and phos-ERK1/2 increased significantly in miR-7^*def*^ BTI model (*P* < 0.05). These results indicate that miR-7 deficiency aggravates the pathology of BTI by altering the transduction of the NF-κB and ERK1/2 signaling pathways.

### RORα is a novel direct target of miR-7 in BTI model

**Fig. 4:**
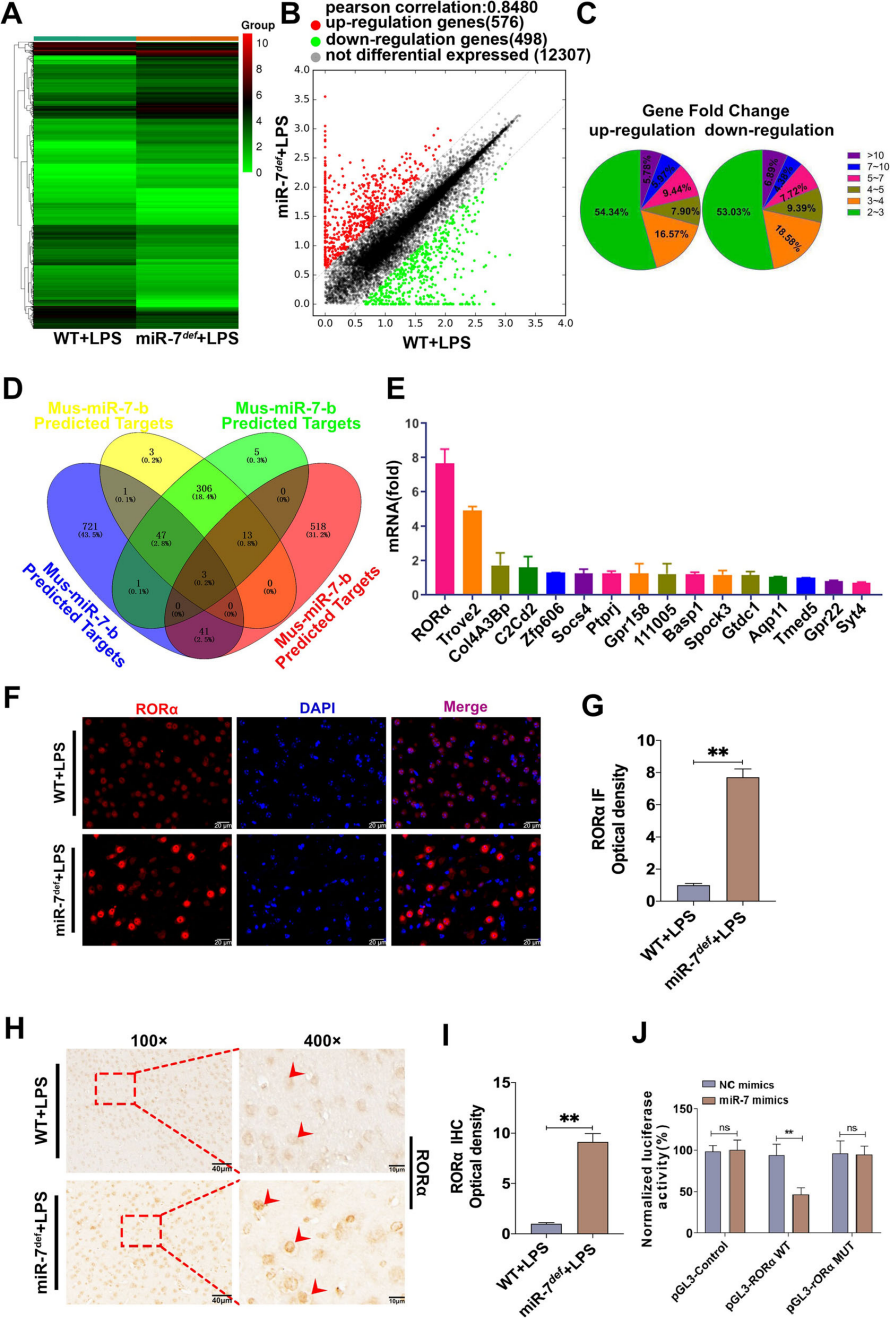
RORα is a novel target of miR-7 in BTI model. WT mice and miR-7^*def*^ mice were intraperitoneally injected with LPS (2.5 mg/kg of body weight). After 12 h, brain tissue was collected. The global gene expression was analyzed by cDNA chip array. **a** Heatmap and **b** scatter plot of gene expression. **c** The fold change and frequency. **d** Prediction of 16 target genes by using miRDB database (http://mirdb.org) and venn analysis. **e** The fold change of the potential target genes of miR-7 in the brain tissue was analyzed by real-time PCR assay and calculated (*n* = 3, one-way ANOVA). **f**, **g** The expression level of RORα was also determined by IF and **h**, **i** IHC assay (*n* = 3, one-way ANOVA). **j** Luciferase assay (*n* = 3, one-way ANOVA, **P < 0.01)

In order to explore the underlying mechanism of miR-7 deficiency on the pathology of BTI, we analyzed the global gene expression profile in brain tissue of BTI model (Fig. 4a, b). As shown in Fig. 4 c, given a twofold change (up and down) in differential expression as a cutoff, 519 genes were upregulated, and 479 genes were downregulated. Then, as shown in Fig. 4 d, we used miRDB database and Venn analysis software to screen out 16 upregulated genes, including RAR-related orphan receptor alpha (RORα), TROVE domain family, member 2(Trove2), Collagen, type IV, alpha 3 (Goodpasture antigen) binding protein (Col4A3Bp), C2 domain-containing protein 2 (C2Cd2), Zinc finger protein 606 (ZFP606), suppressor of cytokine signaling (SOCS4), Receptor-type tyrosine-protein phosphatase eta (PTPRJ), G protein-coupled receptor 158 (GPR158), 111005, Brain abundant membrane attached signal protein 1(BASP1), sparc/osteonectin, cwcv and kazal-like domains proteoglycan 3(SPOCK3), Glycosyltransferase-like domain-containing protein 1(GTDC1), Aquaporin11(Aqp11), transmembrane emp24 protein transport domain containing 5 (TMED5), G protein-coupled receptor22(GPR22), and Synaptotagmin-4 (Syt4), which were closely associated with inflammatory diseases according to previous literatures [34–37]. To further investigate the putative target of miR-7, we verified the expression levels of these 16 predicted target genes, respectively. Notably, in all predicted target genes of miR-7, RORα was significantly upregulated more than eightfold in the brain tissue of miR-7^*def*^ BTI model compared with WT BTI model (Fig. 4e, *P* < 0.05). Furthermore, IF and IHC analysis also showed that the expression level of RORα increased significantly in brain tissue of miR-7^*def*^ BTI model (Fig. 4f–i, *P* < 0.01). Finally, luciferase gene reporter assay showed that miR-7 could directly regulate RORα expression (Fig. 4g). Collectively, these data demonstrate that RORα is a novel target of miR-7 in BTI model.

### RORα is essential for the development of BTI

**Fig. 5:**
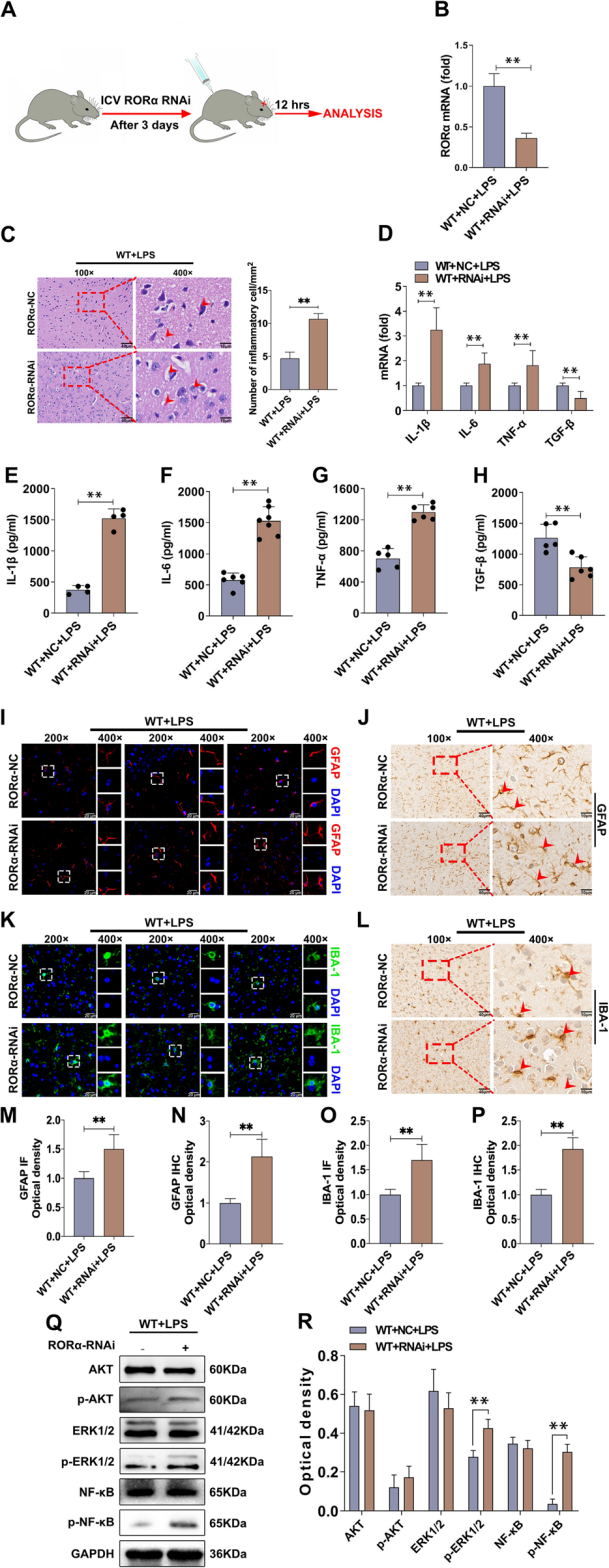
Silence of RORα aggravates the pathology and alters the NF-κB, ERK signaling pathways. **a** The schematic representation of the animal experiments. WT mice (*n* = 6 per group) were transfected with RORα RNAi or negative control (NC) RNAi through lateral ventricle. After 3 days, these mice were intraperitoneally injected with LPS (2.5 mg/kg of body weight). Twelve hours later, brain tissue was collected. **b** The expression level of RORα in brain tissue was analyzed by real-time PCR assay and calculated (*n* = 6 per group, *t* test, **P < 0.01). **c** The pathology of brain tissue was observed by H&E staining (*n* = 3 per group). Arrows in **c** indicate vacuolar degeneration. **d** The mRNA levels of cytokines (IL-1β, IL-6, TNF-α, and TGF-β) were analyzed by real-time PCR assay and calculated (*n* = 5, one-way ANOVA, **P < 0.01). **e**–**h** The protein levels of cytokines were analyzed by ELISA assay and calculated (*n* = 4, one-way ANOVA, **P < 0.01). **i**–**p** The number of astrocytes and microglia were analyzed by IF and IHC and calculated, respectively (*n* = 3, one-way ANOVA, **P < 0.01). **q**, **r** The protein levels of AKT, phos-AKT, ERK1/2, phos-ERK1/2, NF-κB, and phos-NF-κB were analyzed by Western blot assay and calculated (*n* = 3, one-way ANOVA, *P < 0.05, **P < 0.01)

RORα belongs to a member of the NR1 subfamily of nuclear hormone receptors. An increasing body of literature has documented that RORα plays an important role in inflammatory reaction [38–40]. However, the possible role of RORα in the pathology of BTI is still unknown. Then, we silenced the expression of RORα by RNAi to observe the possible change on the pathology of BTI. As shown in Fig. 5 a and b, the relative expression level of RORα reduced significantly in brain tissue from RORα-siRNA-treated group compared with control group (*P* < 0.05). Surprisingly, the number of inflammatory cells and vacuolar degeneration increased obviously in RORα-siRNA-treated group (Fig. 5c). Moreover, the mRNA expression levels of pro-inflammatory factor IL-1β, IL-6, and TNF-α in brain tissue also increased significantly (Fig. 5d, *P* < 0.05). By contrast, the expression level of anti-inflammatory factor TGF-β decreased noticeably (Fig. 5d, *P* < 0.05). Meanwhile, similar results were also obtained by ELISA assay (Fig. 5e–h, *P* < 0.05). IHC and IF assay further showed that the number of astrocytes and microglia elevated significantly in RORα-siRNA-treated group (Fig. 5i–p, *P* < 0.05). Finally, we analyzed the possible change of AKT, ERK, and NF-κB signaling pathways and found that the levels of phos-NF-κB and phos-ERK1/2 in RORα-siRNA-treated group increased significantly (Fig. 5q, r; *P* < 0.05). These results indicate that RORα plays an important role in the pathology of BTI.

### Downregulation of RORα aggravates the pathology of miR-7^*def*^ BTI model

**Fig. 6:**
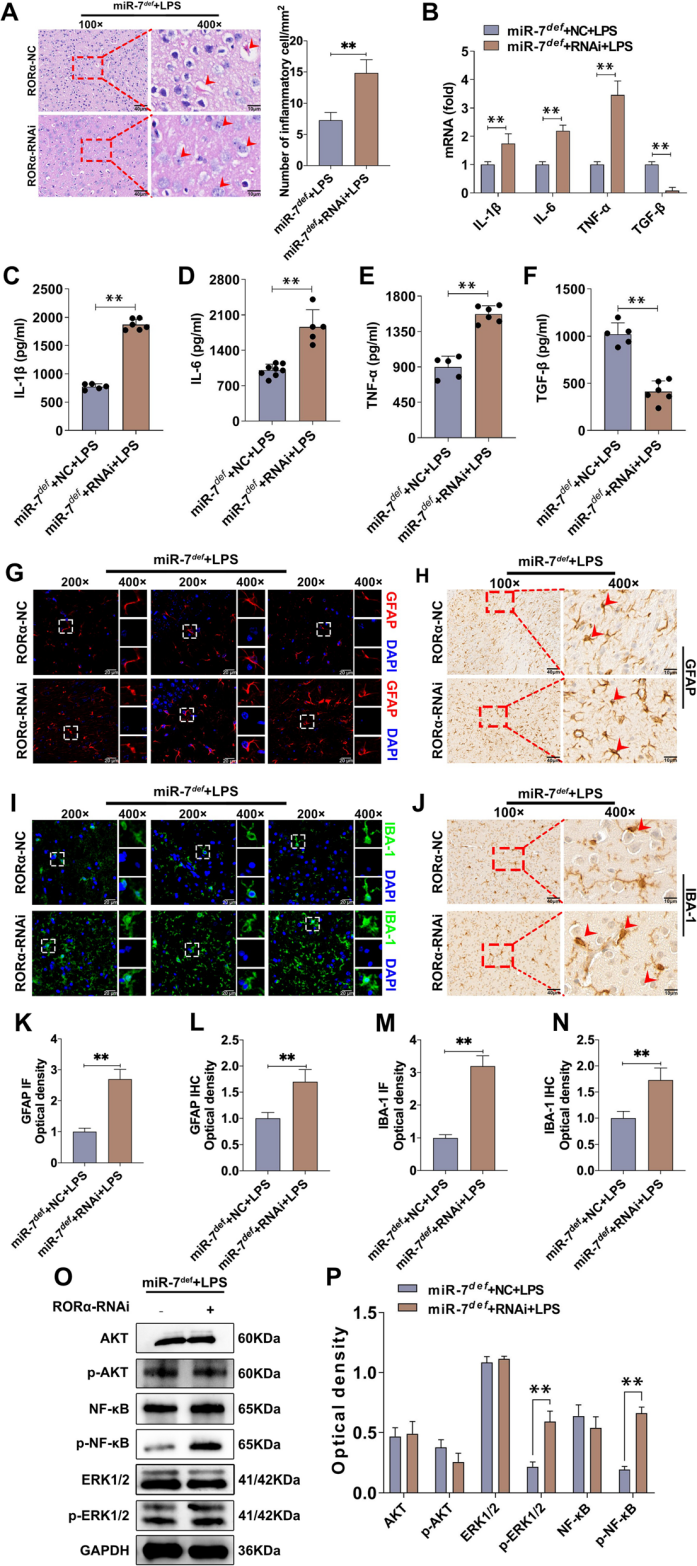
Silence of RORα aggravates the pathology of miR-7^*def*^BTI mice. MiR-7^*def*^ mice (*n* = 6 per group) were transfected with RORα RNAi or NC RNAi through lateral ventricle. After 3 days, these mice were intraperitoneally injected with LPS (2.5 mg/kg of body weight). Twelve hours later, brain tissue was collected. **a** The pathology was observed by H&E (*n* = 3 per group). Arrows in **c** indicate nuclear fragments. **b** The mRNA levels of cytokines (IL-1β, IL-6, TNF-α, and TGF-β) were analyzed by real-time PCR assay and calculated (*n* = 6, one-way ANOVA, **P < 0.01). **c**–**f** The protein levels of cytokines were determined by ELISA assay and calculated (n = 5, one-way ANOVA, **P < 0.01). **g**–**n** The number of astrocytes in hippocampus and microglia in cerebral cortex were analyzed by IF and IHC (*n* = 3, one-way ANOVA, **P < 0.01). **o**, **p** The protein levels of signaling molecules including AKT, phos-AKT, ERK1/2, phos-ERK1/2, NF-κB, and phos-NF-κB in brain tissue were analyzed by Western blot assay and calculated (*n* = 3, one-way ANOVA, **P < 0.01)

To further explore the underlying role of RORα in the effect of miR-7 deficiency on BTI, we silenced the expression of RORα in miR-7^*def*^mice and then BTI model was constructed as in the above description. The number of inflammatory cells and nuclear fragments increased obviously in brain tissue in RORα-siRNA-treated group (Fig. 6a). Moreover, as shown in Fig. 6 b, the relative expression levels of pro-inflammatory factor TNF-α, IL-1β, and IL-6 increased significantly in RORα-siRNA-treated group while anti-inflammatory factor TGF-β decreased noticeably compared with control group (*P* < 0.05). Meanwhile, similar results were obtained by ELISA assay (Fig. 6c–f, *P* < 0.05).

Furthermore, IHC and IF assay showed that the number of astrocytes and microglia were elevated significantly in RORα-siRNA-treated group (Fig. 6g–n). Finally, we analyzed the possible change of AKT, ERK, and NF-κB signaling pathways. As shown in Fig. 6 o and p, the protein levels of phos-NF-κB and phos-ERK1/2 in RORα-siRNA-treated group increased significantly (*P* < 0.05). These results demonstrate that downregulation of RORα exacerbates the effect of miR-7 deficiency on the pathology of BTI.

### RORα and miR-7 are co-expressed in neurons in BTI model

**Fig. 7:**
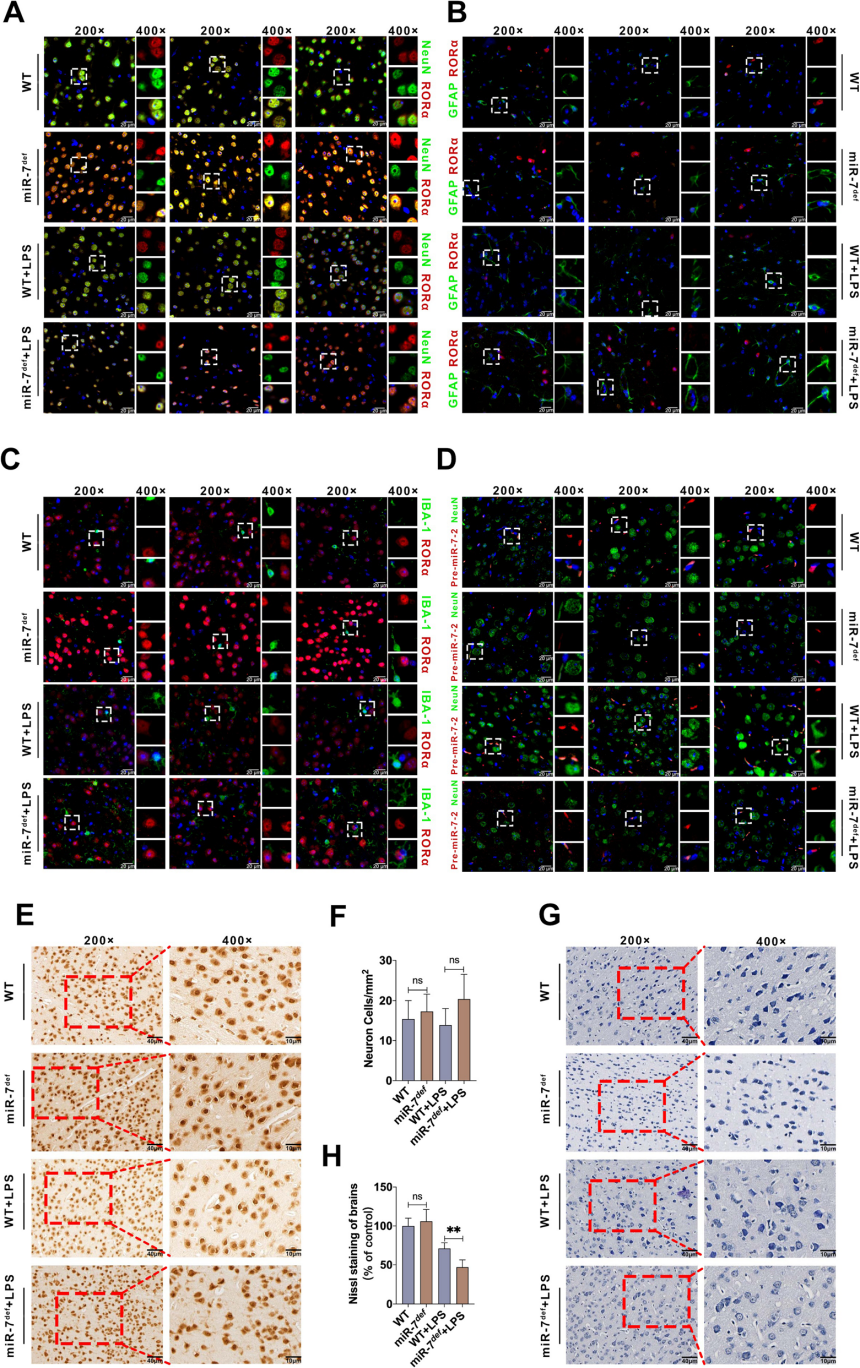
RORα and miR-7 were co-expression in the neuron. WT mice and miR-7^*def*^ mice (*n* = 6 per group) were intraperitoneally injected with LPS (2.5 mg/kg of body weight) and control group with PBS, respectively. After 12 h, **a**–**c** the colocated expression of RORα and NeuN, GFAP or IBA-1 were detected by double immunofluorescence labeling assay. **d** The expression of pre-miR-7-2 in neuron was analyzed by IF and FISH assay (*n* = 6 per group). **e**–**h** The change of neuron and Nissl bodies were detected by IHC and Nissl staining, respectively (*n* = 3, one-way ANOVA, **P < 0.01)

Next, in order to further explore the connection between RORα and miR-7 in pathology of BTI, we analyzed the expression of RORα and miR-7 in neurons, microglia, and astrocytes, which are major cell populations in brain tissue in BTI model. Interestingly, double immunofluorescence labeling assay showed that RORα was dominantly expressed in neurons of brain tissue (Fig. 7a–c). However, miR-7 expressed both in neurons and other cells (Fig. 7d). Importantly, in neurons, the expression level of RORα increased obviously in miR-7^*def*^ BTI model compared with WT BTI model (Fig. 7a), which was consistent with our above data.

Previous studies have shown that neurons are involved in the pathology of BTI [41–44]. And we found that RORα and miR-7 were co-expressed in neurons of BTI model. Then, we further observed the possible change of neurons in brain tissue from BTI model. Data showed that, compared with WT group, the number of neuron cells did not change significantly in the brain tissue in WT BTI model group (Fig. 7e, f, *P* > 0.05). However, we found that, compared with WT group, the number of Nissl bodies in neurons of WT BTI model group decreased obviously (Fig. 7g, h, *P* < 0.05), which was consistent with previous literature [45]. Most importantly, compared with WT BTI model, the number of Nissl bodies in neurons decreased obviously in miR-7^*def*^ BTI model (Fig. 7g, h, *P* < 0.05), indicating that miR-7 deficiency might impair the physiologic function of neurons.

### RORα synergizes with miR-7 to control the inflammatory reaction of neuronal cells in vitro

**Fig. 8:**
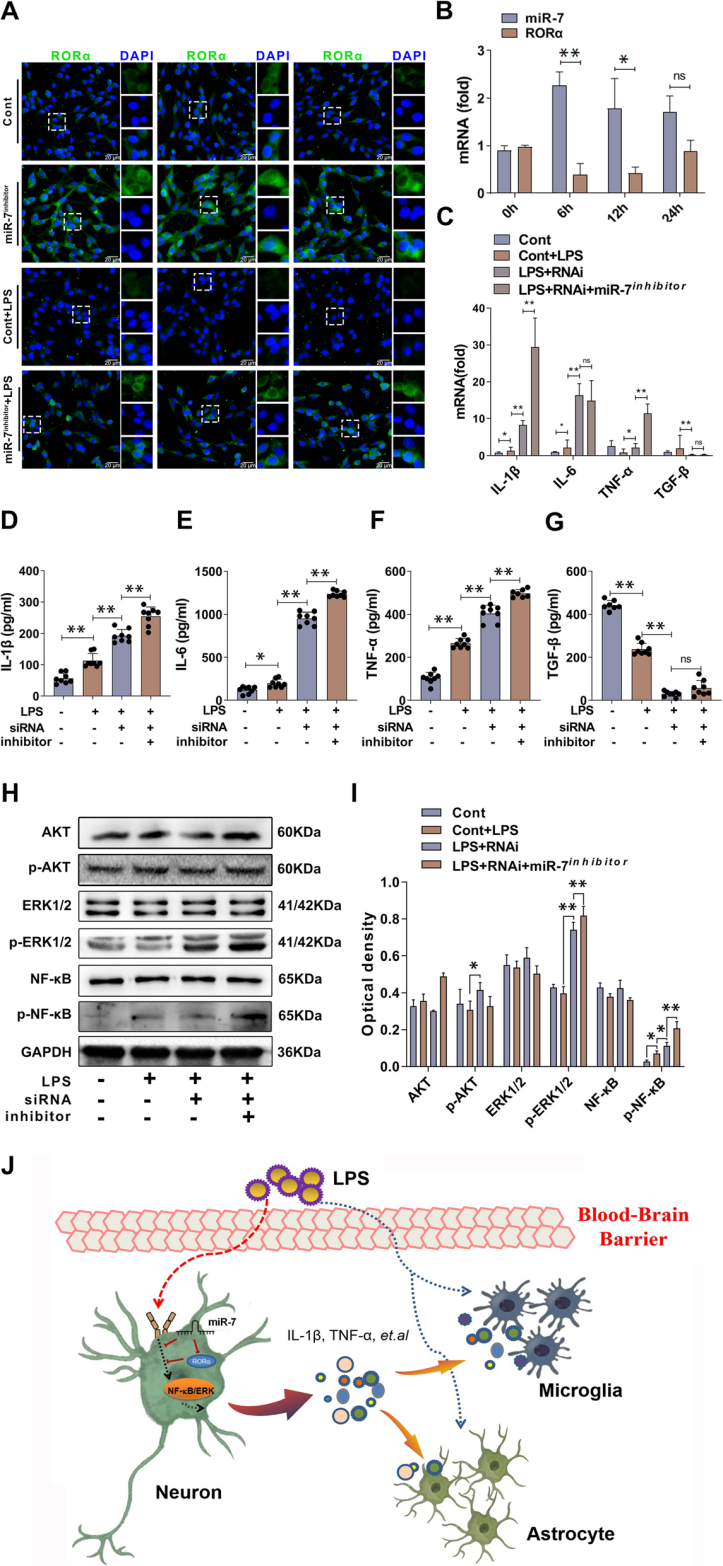
RORα synergizes with miR-7 to control the inflammatory reaction of neuronal cells in vitro. **a** Neuronal cell PC12 cells were stimulated by 100 ng/mL LPS for 12 h, the expression level of RORα was determined by double immunofluorescence labeling assay. **b** The expression levels of miR-7 and RORα in neuronal cells were determined by real-time PCR assay and calculated. Neuronal cells were infected with RORα RNAi or NC and then stimulated by 100 ng/mL LPS for 12 h, **c**–**g** the expression levels of cytokines (IL-1β, IL-6, TNF-α, and TGF-β) were determined by real-time PCR assay and ELISA assay (*n* = 7, one-way ANOVA, *P < 0.05, **P < 0.01). **h**, **i** The protein levels of signaling molecules including AKT, phos-AKT, ERK1/2, phos-ERK1/2, NF-κB, and phos-NF-κB were determined by Western blot assay and calculated (*n* = 3, one-way ANOVA; *P < 0.05, **P < 0.01). **j** Schematic representation of the underlying mechanism of miR-7 and its target molecule RORα synergistically controlled the inflammatory reaction of neurons in response to LPS exposure, which subsequently affected the function of other cells including microglia and astrocytes and ultimately orchestrated the development of BTI

Finally, in order to explore the role of RORα and miR-7 in inflammatory reaction of neurons, we observe the expression of RORα and miR-7 in neuronal cells in response to LPS stimulation. Data showed that, compared with that in control group, the expression level of RORα increased significantly in neuronal cells in miR-7 inhibitor-transfected group (Fig. 8a, *P* < 0.05). Importantly, we found that compared with that in LPS-treated group, the expression level of RORα increased dramatically in LPS-treated miR-7 inhibitor-transfected group (Fig. 8a, *P* < 0.05), which was consistent with our above data. Furthermore, we found that, after LPS stimulation, the expression of miR-7 in neuronal cells increased rapidly to the peak at 6 h and then gradually decreased (Fig. 8b), while the expression of RORα decreased significantly at 6 h and then steadily increased, displaying a contrary expression pattern to miR-7 (Fig. 8b).

Next, we detected the production of inflammatory cytokines in neuronal cells in response to LPS stimulation and found that, as shown in Fig. 8 c, the expression levels of pro-inflammatory factor TNF-α, IL-1β, and IL-6 increased significantly in RORα-RNAi-transfected group compared with control group (*p* < 0.01). As expected, compared with those in RORα-RNAi-transfected group, the expression levels of pro-inflammatory factor TNF-α, IL-1β, and IL-6 increased obviously in RORα-RNAi plus miR-7 inhibitor co-transfected group (Fig. 8c, *P* < 0.05). Conversely, the expression level of anti-inflammatory factor TGF-β decreased noticeably (Fig. 8c, *P* < 0.05). To verify these data, we detected the protein levels of these cytokines by ELISA assay and obtained similar results (Fig. 8d–g, *P* < 0.05). Finally, we also analyzed the possible change in AKT, ERK, and NF-κB signaling pathways. Data showed that the levels of phos-NF-κB and phos-ERK1/2 in RORα-RNAi-transfected group increased significantly compared with those in control group (Fig. 8h, i, *P* < 0.05). Importantly, we found that the levels of phos-NF-κB and phos-ERK1/2 in RORα-RNAi plus miR-7 inhibitor co-transfected group increased significantly compared with those in RORα-RNAi-transfected group (Fig. 8h, i, *P* < 0.05). Together, these observations suggested that RORα synergizes with miR-7 to control the inflammatory reaction of neuronal cells, which is closely correlated with the altered transduction of the NF-κB and ERK signaling pathways.

## Discussion

Up to now, this is the first study to explore the potential role of miR-7 in the pathology of BTI. Herein, we found that the expression level of miR-7 increased significantly in LPS-induced BTI model. Furthermore, miR-7 deficiency could aggravate the pathology of BTI. Importantly, the expression level of RORα, a novel target of miR-7, was upregulated in brain tissue of BTI model with miR-7 deficiency. Unexpectedly, silence of RORα remarkably exacerbated, but not alleviated, the pathology of brain tissue, as well as promoted the transduction of NF-κB and ERK1/2 signaling pathways in BTI model with or without miR-7 deficiency. Finally, RORα and miR-7 were dominantly co-expressed in neurons from BTI mice and synergistically controlled the inflammatory reaction of neuronal cells in response to LPS stimulation.

Recently, miR-7 has been reported to play an important role in regulating the biological process of various diseases [46, 47]. For instance, miR-7 is a tumor suppressor and increase cisplatin sensitivity of gastric cancer cells by targeting mTOR, indicating its potential application for the treatment of human gastric cancer in the future [48]. Moreover, miR-7 can regulate α-synuclein expression and downregulation of miR-7 results in the loss of dopaminergic neuronal in the substantia nigra [49]. In our previous study, miR-7 can regulate the pathology of acute lung injury [23]. Herein, we extended these previous findings by demonstrating that miR-7 was upregulated in BTI model. Importantly, miR-7 deficiency could aggravate the pathology of brain tissue, indicating miR-7 was a novel negative intrinsic regulator in BTI. Similarly, Cao’s study found that long noncoding RNA small nucleolar RNA host gene 1 promoted neuroinflammation in the pathogenesis of Parkinson’s disease via modulating miR-7/NLRP3 pathway [18]. Zhang’s study found that miR-7 inhibited the expression of TLR4 and reduced LPS-induced inflammatory response produced by microglial cells and then alleviated the inflammation in the brain of rats with cerebral hemorrhage [50]. It is well known that inflammation is a common pathological basis for various neurological diseases; therefore, our current findings might not only aid the understanding on the role of miR-7 in pathogenesis of BTI, but also provide a valuable clue for the development of therapeutic strategies against neurological diseases.

*RORα* localizes on chromosome 9q22.2. Recent studies have shown that RORα plays a vital role in the inhibition of the NF-κB signaling pathway transduction [51, 52]. It is well known that NF-κB pathway is critical for the development of inflammatory response [53, 54], indicating that RORα might be a suppressor in inflammation. Reyes-Gibby et al. found that RORα might be a novel target gene by analyzing single nucleotide polymorphisms in patients with neuropathic pain, thereby providing a new therapeutic strategy for treatment and management of neuropathic pain [55]. In the present study, the expression level of RORα increased in brain tissue in miR-7 deficiency BTI model mice. Importantly, the repression of RORα could exacerbate the pathology of BTI, accompanied by elevated transduction of the NF-κB signaling pathway. Consistently, literature documented that RORα could repress the transduction of NF-κB signaling pathway [56]. Therefore, combining these data demonstrated the important role of RORα/NF-κB axis in the pathology of BTI. Interestingly, we noticed that the repression of RORα also altered the transduction of ERK1/2 signaling pathway. There is a growing body of evidence that suggests there is connection between NF-κB pathway and ERK1/2 pathway [57–59]. Even though, the exact role of RORα in ERK1/2 pathway still needs to be investigated in successive research work, which is important for the exploration of the pathogenesis of BTI and related inflammation brain diseases.

In biological events, synergism and antagonism effect are main forms of different genes in bringing into play biological function. Numerous studies have documented that the network among miRNA molecules and their target genes is complex and critical for the development of various diseases, in which miRNA usually controlled the expression of their targets [60, 61]. However, whether there is synergy effect between miRNAs and target genes still remains unknown. In our study, we found that RORα was a new target of miR-7 in BTI. Surprisingly, unlike previous reports that miRNAs exerted opposite biological function to their targets, repression of RORα could aggravate, but not reverse, the pathology of BTI in the condition of miR-7 deficiency, indicating that RORα exerted negative regulation on pathology of BTI in the absence of miR-7. Given the fact that RORα expression was upregulated when miR-7 was deficient, then, our current study might raise a new network model in which miR-7 could control the pathology of BTI, synergistically with its target RORα. To this interesting phenomenon, we proposed it reflected the complexity of network among miRNA and their targets in the development of various diseases. Finally, it also would be pointed out that other targets, through which miR-7 controls the pathology of BTI, were not investigated in current study and remain to be elucidated in the future. Therefore, further investigation on the expression patterns of miR-7 and its different targets during the development of BTI is valuable for the validation of connections among miR-7 and its multiple targets in the pathology of BTI.

Accumulating evidence has shown that the change of biological function of neuronal cells is involved in the development of various brain diseases [62, 63]. Moreover, the relationship among neurons and other cells such as microglia and astrocytes is complex in the pathology of brain diseases [64, 65]. For instance, La et al. found that alpha-synuclein oligomers activating glial cells led to neuron damage and thus were emerging as crucial factors in the pathogenesis of synucleinopathies [66]. Wang et al. reported that HAPLN2 involved in the pathogenesis of schizophrenia by regulating the neuron migration and velocity of nerve conduction [67]. Similarly, Komura et al. found that amyl spheroids were accumulated mainly in the trans-golgi network of excitatory neuronal cells, causing the degeneration of adjacent NAKα3-expressing neurons in Alzheimer’s disease [68]. In the present study, we found that the number of microglia and astrocytes were elevated in BTI model with miR-7 deficiency. Moreover, the physiologic function of neurons was also impaired. However, we revealed that RORα was dominantly expressed in neurons, but not in microglia and astrocytes. Of note, miR-7 and RORα were also co-expressed in neurons. Furthermore, in the absence of miR-7, inhibition of RORα promoted the production of pro-inflammatory cytokines of neurons in response to LPS, which was closely correlated with the altered transduction of the NF-κB and ERK signaling pathways. In line with these finding, some studies have documented that LPS can stimulate neurons to secrete pro-inflammatory cytokines, which activate microglia, astrocytes, and other immune cells [69–71]. Therefore, our current data further support the important relationship among neurons and other cells in brain diseases, which might benefit the exploration on the cellular mechanism of the development of inflammation brain diseases.

## Conclusions

Taken together, we found that miR-7 and its target molecule RORα synergistically controlled the inflammatory reaction of neurons, which subsequently affected the function of other cells and ultimately orchestrated the pathology of BTI (Fig. 8j). Importantly, our findings might provide a new light on the network among miRNAs and their targets and aid the mechanistic understanding on BTI development, as well as the progression of new therapeutic strategies against clinical inflammatory brain diseases.

## Data Availability

All data are available upon request.

## Abbreviations

3′-UTR: 3′-untranslated region
BTI: Brain tissue inflammation
def: Deficiency
miR-7: MicroRNA-7
RORα: RAR-related Orphan Receptor Alpha
